# Thermal patterns in zoster

**Published:** 2015

**Authors:** IM Cojocaru, MC Cojocaru, VM Voiculescu, OB Bozdoc-Ionescu, AM Cartog, C Giurcaneanu

**Affiliations:** * Department of Dermatology, Elias University Hospital, Bucharest, Romania; ** Department of Rehabilitation Medicine, Elias University Hospital, Bucharest, Romania; *** ”Carol Davila” University of Medicine and Pharmacy, Bucharest, Romania; **** Polimed Clinic, Bucharest, Romania; ***** Department of Dermatology, Clinical and Emergency Hospital, Bucharest, Romania

**Keywords:** varicella zoster virus, neuropathic pain, skin changes, inflammation, thermography

## Abstract

**Rationale:** Varicella zoster virus is a neurotropic virus that causes an infectious disease characterized by skin changes and neuropathic pain. After the resolution of the first infection, the virus lies dormant within the sensory ganglia. The reactivation of the virus causes zoster. An alteration in skin infrared emission might be expected in the areas of the skin affected by inflammatory changes and demyelination of the affected peripheral nerve.

**Objective:** To establish the importance of thermal imaging in the follow up of Zoster Zone with different localization. An infrared thermal camera was used in order to assess if the evolution of the disease determines a thermal pattern.

**Methods and Results:** Infrared thermography can be used for the assessment of the affected area also by using a thermography camera that is sensitive to the infrared spectrum.

An intense and diffuse infrared emission is highly suggestive for the inflammation and implies that a more aggressive treatment should be initiated. After the clinical resolution of the affected area, the symmetry of the thermal pattern should be restored. If the asymmetry persists, a neuropathic complication of the virus reactivation could be involved.

**Discussions:** The integration of infrared thermography with the clinical findings is very useful in order to create a complete picture of the zoster lesions and this method could determine the beginning of a correct treatment and, by doing so, minimizing the risk of complications.

## Introduction

Varicella zoster virus is a neurotropic virus that causes an infectious disease characterized by skin changes and neuropathic pain. After the resolution of the first infection, the virus lies dormant within the sensory ganglia. The reactivation of the virus causes zoster. An alteration in skin infrared emission might be expected in the areas of skin affected by inflammatory changes and demyelination of the affected peripheral nerve. The most important risk for the development of zoster is age: almost 50% of the persons over 85 years old experience an episode of zoster [**1**]. The main factors that trigger zoster are rheumatoid arthritis (2.1% vs. 1.5%), inflammatory bowel disease (1.3% vs. 0.9%), chronic obstructive pulmonary disease (4.7% vs. 3.7%), asthma (7.1% vs. 5.8%), chronic kidney disease (6.0% vs. 5.4%), depression (4.7% vs. 4.0%) [**2**]. Most of the patients with zoster experience pain due to inflammation of the peripheral nerve and the skin rash. The pain that lasts over three months after the skin rash disappears is called post-herpetic neuralgia (PHN). The development of PHN is associated with high pain intensity in the acute phase, patients’ age and the immunological status [**3**,**4**].

Thermography in infrared spectrum is a tool useful for the determination of the variations in temperature of the different areas of the skin. In the areas of skin affected by inflammatory changes and demyelination of the affected peripheral nerve, an alteration in skin infrared emission might be expected. The affected area can be warmer or cooler [**5**-**7**]. The aim of this study was to determine the thermal patterns of different zoster localizations.

## Material and Methods

12 patients (4 females and 8 males, with ages 28-85 years) suffering from acute herpes zoster were included in this study. The disease was clinically identified for each patient and after that, a thermal image of the affected area was taken. The following information could be collected from each patient: gender, age, time of onset, and pain intensity measured by VAS (visual analog scale). VAS is a scale from 0 to 10, 0 representing no pain and 10 representing the worst pain ever. The onset of the herpes skin lesions was 1 to 21 days prior to thermographic investigation. The affected dermatomes are shown in **Table 1**.

Four out of 12 patients were reexamined (clinically, thermographic and pain intensity by VAS) one month later (**Table 1**, VASr column). 

Thermography was performed by using a Trotec® EC 60 camera with a 160x120 pixels detector and a spectral range from 8 to 14 µm. The images acquired with the camera were analyzed by using IC IR-Report pro® software which was provided with the camera. The thermography was performed by a single examiner, after the patient rested for 15 minutes in a room without direct sunlight or incandescent light and the temperature of the environment of 23°C.

We used the Line tool (L followed by a number) to evaluate the temperature distribution across a vertical line through the body area, from left to right, intersecting the area affected by zoster.

The patients were selected from multiple clinics and all of them had signed the appropriate informed consent.

**Table 1:** Affected region with the corresponding dermatome (VASr = VAS scores at reevaluation)

Affected region	Dermatomes	Number of patients	VAS	VASr
Face	Trigeminal	1	2	
Cervical spine	C2	1	3	
	C4 - C5	1	5	
	C6	1	5	
	C5 - T1	1	8	2
Thoracic spine	T2 - T3	3	7	
			10	2
			10	
	T3 - T4	1	9	0
Lumbar spine	L1 – L2	1	5	1
	S1 – S2	2	1	
			10	

## Results

A patient presented the skin rash on his face, in the trigeminal area, one patient had herpetic changes in the nuchal region (C2) and, in 3 patients, the localization was at C4-C5-C6 dermatomes level. The other 7 patients presented thoracic or lumbar involvement: 4 patients at the thoracic level (T2-T3-T4) and 3 patients at the lumbar level.

**Fig. 1:**
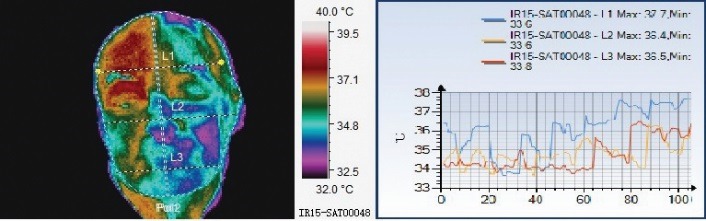
The patient with zoster; all of the lines show an increase in temperature

It could be observed that the affected side had a different thermal pattern than the healthy side, the patients presenting higher temperatures on the affected side. The entire evolution of Herpes Zoster is marked by dynamic changes in skin temperature [**8**,**9**]. As a clinical observation for the patient with facial zoster, all the areas of the right side of the face innervated by the facial nerve had a higher average temperature than the corresponding areas of the left side of the face and the right orbito-fronto-temporal area (the place with herpes zoster lesions) had an even higher temperature (**Fig. 1**). 

**Fig. 2:**
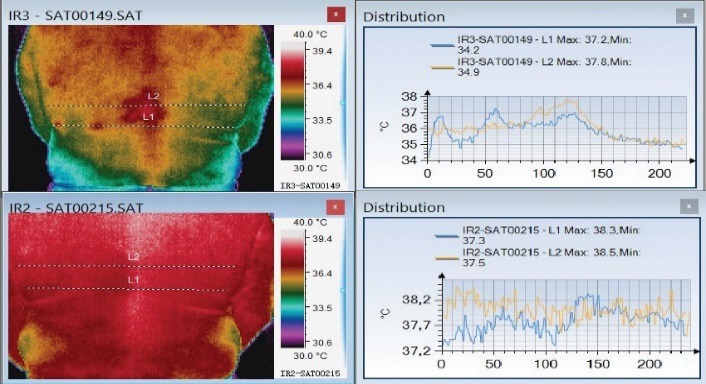
Thoracic Herpes zoster with intense pain in the acute phase. The thermal symmetry restores one month later. In the initial phase, L1 and L2 representation had spikes corresponding to the lesion. Those spikes are no longer visible one month later

**Fig. 3:**
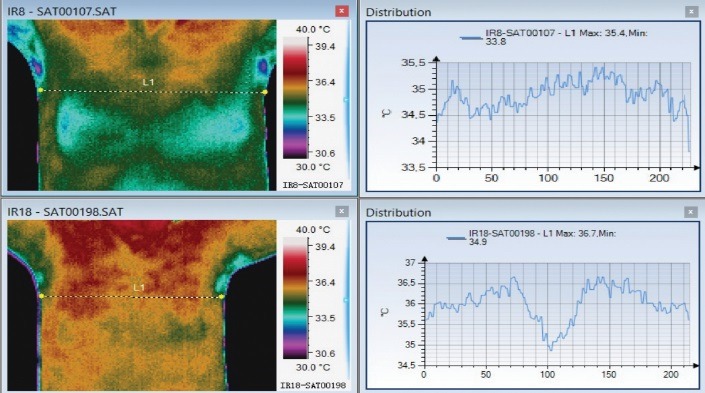
Thoracic herpes zoster in the acute phase (above) and one month later (below). In the acute phase, the affected area is warmer and then thermal symmetry is reestablished one month later

**Fig. 4:**
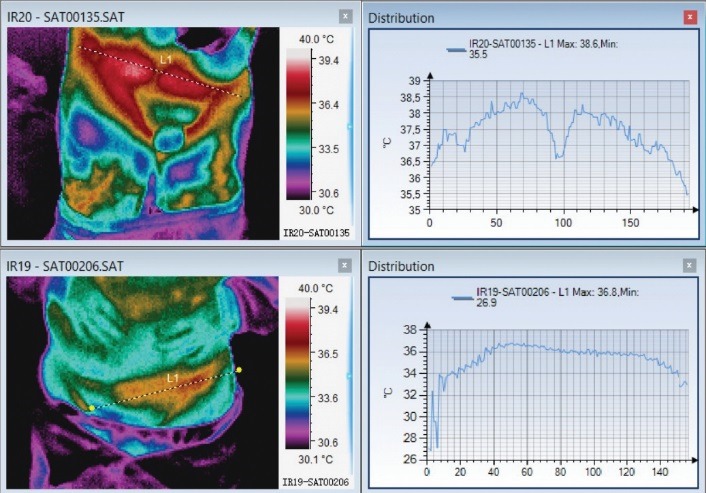
Herpes Zoster at L1–L2 dermatomes level (right groin area)

**Fig. 5:**
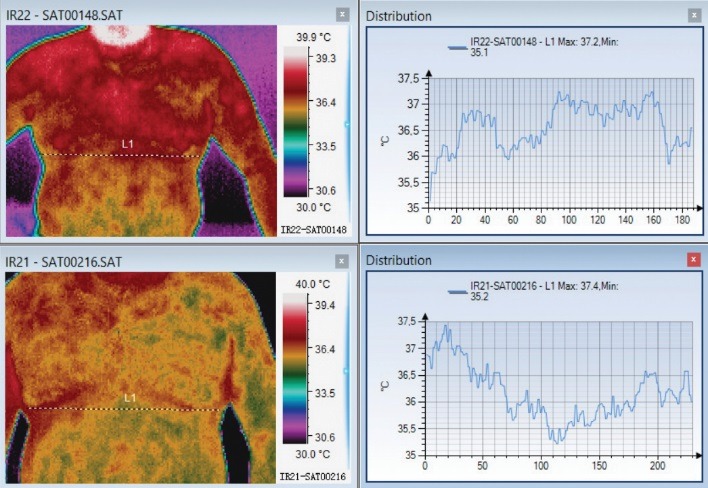
Thoracic Herpes Zoster – acute phase and remission. Thermal asymmetry can be detected in both thermal pictures

**Fig. 6:**
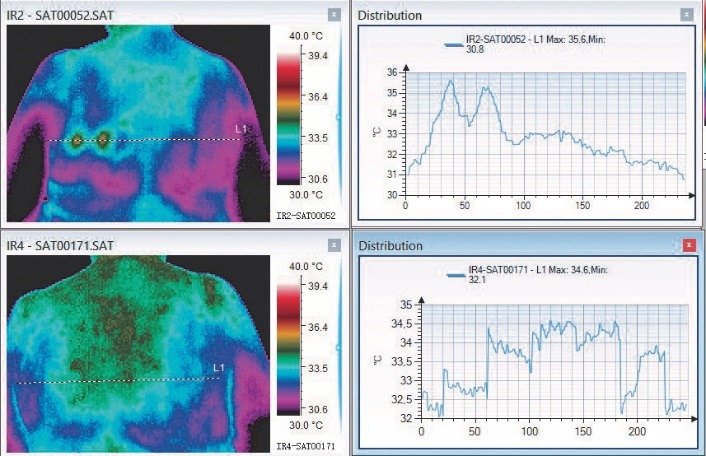
Thoracic herpes zoster with thermal asymmetry in both acute and remission phase

In the acute phase, the affected areas are warmer (as seen in **Fig. 1**-**6**) and after the remission, the thermal symmetry should be restored (**Fig. 2**, **3**). However, thermal asymmetry after herpes zoster can be detected during the follow-up process. Colder or warmer patterns are present in **Fig. 4**-**6**.

A correlation between changes in skin temperature in the acute phase and VAS scores could not be made, but a high initial VAS score correlates with the persistence of thermal asymmetry one month later.

In **Fig. 4**, the above thermal image was made during the acute phase. The side affected by herpetic changes is warmer. In this phase, the VAS score was 5. Below, the image was acquired during the one-month reevaluation. The area prior affected by the disease shows a colder pattern. Even if the initial VAS score was not high, the patient maintained a postherpetic neuralgia (VAS score 1).

**Fig. 5** represents a patient with thoracic herpes zoster, who presented a very high VAS score [**10**] with thermal asymmetry (above) in the acute phase and one month later (below), thermal asymmetry can still be detected on the ventral region and the patient maintained a score of 2 on VAS scale. The affected area is colder than the healthy one.

In the initial phase, the two spikes corresponding to the active lesion could be observed and after one month, it could be observed that the average temperature on the affected side was higher. VAS score suffered a decrease from 10 initially to 2 after one month. 

## Discussions

Thermal imaging has its limitation in medical practice. The method cannot see more than the temperature of the skin, and needs special protocol and it is unreliable as a stand-alone investigation for the diagnosis of herpes zoster. It would be interesting to use thermography for the diagnosis of herpes zoster sine herpete or for the diagnosis of herpes zoster before the skin changes appear (when pain appears before the rash) [**12**]. The same thermal pattern can be seen in a localized infection of the skin, so the clinical assessment is very important.

## Conclusions

Infrared thermography could be a great asset for the Dermatology Department in monitoring the conditions associated with tissue temperature alterations.

The integration of infrared thermography with the clinical findings is very useful in order to create a complete picture of the zoster lesions. It is a fast, real time, reliable, non-invasive and non-ionizing method that can be used as a high tech auxiliary diagnostic method [**11**,**12**].

**Acknowledgement**

This paper is supported by the Sectorial Operational Programme Human Resources Development (SOP HRD), financed from the European Social Fund and by the Romanian Government under the contract number POSDRU/159/1.5/S/137390.

**Disclosures**

None.
